# Intraoperative pelvic neuromonitoring based on bioimpedance signals: a new method analyzed on 30 patients

**DOI:** 10.1007/s00423-024-03403-y

**Published:** 2024-08-03

**Authors:** Georgi Kalev, Ramona Schuler, Andreas Langer, Matthias Goos, Marko Konschake, Thomas Schiedeck, Christoph Marquardt

**Affiliations:** 1 Department of General, Visceral, Thoracic and Pediatric Surgery, Ludwigsburg Hospital, Posilipostraße 4, 71640 Ludwigsburg, Germany; 2 Department of Surgery, Medical Faculty Mannheim, Universitätsmedizin Mannheim, Heidelberg University, 68167 Mannheim, Germany; 3 Research and Development, Dr. Langer Medical GmbH, 79183 Waldkirch, Germany; 4 Institute of Biomedical Engineering and Informatics, TU Ilmenau, 98693 Ilmenau, Germany; 5 Department of General and Visceral Surgery, Helios Hospital Müllheim, Heliosweg 1, 79379 Müllheim, Germany; 6 Department of Anatomy, Histology and Embryology, Institute of Clinical and Functional Anatomy, Medical University of Innsbruck (MUI), Innsbruck, Austria

**Keywords:** Low anterior resection, Rectal cancer, Neuromonitoring, Pelvic autonomic nerves

## Abstract

**Purpose:**

Increasing importance has been attributed in recent years to the preservation of the pelvic autonomic nerves during rectal resection to achieve better functional results. In addition to improved surgical techniques, intraoperative neuromonitoring may be useful.

**Methods:**

This single-arm prospective study included 30 patients who underwent rectal resection performed with intraoperative neuromonitoring by recording the change in the tissue impedance of the urinary bladder and rectum after stimulation of the pelvic autonomic nerves. The International Prostate Symptom Score, the post-void residual urine volume and the Low Anterior Resection Syndrome Score (LARS score) were assessed during the 12-month follow-up period.

**Results:**

A stimulation-induced change in tissue impedance was observed in 28/30 patients (93.3%). In the presence of risk factors such as low anastomosis, neoadjuvant radiotherapy and a deviation stoma, an average increase of the LARS score by 9 points was observed 12 months after surgery (*p =* 0,04). The function of the urinary bladder remained unaffected in the first week (*p =* 0,7) as well as 12 months after the procedure (*p =* 0,93).

**Conclusion:**

The clinical feasibility of the new method for pelvic intraoperative neuromonitoring could be verified. The benefits of intraoperative pelvic neuromonitoring were particularly evident in difficult intraoperative situations with challenging visualization of the pelvic nerves.

## Introduction

Besides oncological outcomes, patient safety and specifically the preservation of the quality of life, are the focus of modern rectal surgery. Pelvic autonomic nerve preservation (PANP) by respecting the „holy plane“ [1] addresses quality of life preservation, in terms of continence (fecal and urinary) and sexual function. Especially for obese patients, a narrow male pelvis, and patients with bulky tumors nerve-preserving surgery becomes demanding [2, 3]. Furthermore, multivisceral resections have a high risk of nerve damage.

In thyroid surgery, intraoperative neuromonitoring is based on recording electromyographic (EMG) signals generated promptly after nerve stimulation, which enables the identification and mapping of the recurrent laryngeal nerve and external branch of the superior laryngeal nerve. In comparison, stimulation of the pelvic autonomic nerves during pelvic neuromonitoring triggers action potential salvos resulting in a slow modulation of smooth muscle activity, which is rated as an indicator for intact nerves. Although there are significant differences in signal detection between the two technical modalities, the clinical setup is similar - the nerves in the surgical field are directly stimulated by the surgeon with a handheld probe and the targeted organ’s response is documented by a recording of electrophysiological signals [4, 5]. The only commercially available system for pelvic neuromonitoring records electromyography (EMG) on the internal anal sphincter (IAS), and bladder manometry, while the surgeon directly stimulates the pelvic nerves in the surgical field [6–8]. Each measurement of bladder manometry requires bladder filling that lasts a few minutes, which is of minor importance given that the surgical procedure lasts several hours. However, if more frequent stimulation is required, the intraoperative workflow is repeatedly interrupted.

To avoid time-consuming measurements, a new method recording bioimpedance signals on the urinary bladder and the rectum during direct nerve stimulation in the surgical field was carried out after a preclinical animal study involving twelve pigs [5, 9].

In this study, we investigated the clinical feasibility of intraoperative pelvic neuromonitoring in thirty patients. We aimed to evaluate functional clinical outcomes related to defecation habits and urinary voiding in patients who underwent anterior rectal resection.

## Materials and methods

A total of 30 consecutive patients of legal age and legally competent (ASA 1–3) who underwent rectal resection for diverticulitis or rectal cancer, Miles procedure for rectal cancer or resection rectopexy for rectal prolapse with the use of intraoperative pelvic neuromonitoring between December 2020 and September 2022 were enrolled in this prospective monocentric single-arm study. Exclusion criteria were missing consent, pregnancy, patients with active implants (e.g. pacemaker or ICD), patients with epilepsy or severe cardiac arrhythmia, patients who took part in another study in the last 30 days, that could interfere with our study, and patients depending on the sponsor or the examiner. All patients underwent surgery at Ludwigsburg Hospital, Germany. This study was approved by the local Ethics Committee of the State Medical Chamber of Baden-Württemberg (Application No. 00011915/00054594) and the Federal Institute for Drugs and Medical Devices (Application No. 94.1.12-5660-11914). The study followed the ethical standards of the Declaration of Helsinki and was conducted by the principles of good clinical practice (DIN EN ISO 14,155). Written informed consent was obtained from all participants or their legal guardians.

To assess the functional outcomes after low anterior rectal resection, preoperatively, in the first week postoperatively, and after 3, 6, and 12 months the International Prostate Symptom Score (IPSS) [10], the post-void residual urine volume (PVR) and the Low Anterior Resection Syndrome Score (LARS) [11] were determined. Our experienced ostomy nurses collected the continence data using the questionnaires mentioned above. This was done personally at the hospital and later by mail or telephone interviews, if patients were not willing to come personally.

The International Prostate Symptom Score (IPSS) is a tool that can be used to assess the severity of lower urinary tract symptoms after rectal resection. Developed by the American Urological Association and first introduced in 1992 [10], it is an internationally validated, reproducible scoring system consisting of 7 questions related to voiding symptoms. A score of 0 to 7 points refers to mild symptoms, 8 to 19 points to moderate symptoms, and 20 to 35 points to severe symptoms.

The post-void residual urine volume (PVR) was measured with an automated ultrasound method (Bladderscan Prime Plus, Verathon Inc. Bothell, USA) with an accuracy of ± 7.5% on volumes greater than 100 ml and ± 7.5 mL on volumes less than 100 ml).

For the assessment of bowel dysfunction after low anterior rectal resection (so-called Low Anterior Resection Syndrome) we applied the LARS score [11]. The questionnaires contain the 5 following items: “incontinence for flatus,” “incontinence for liquid stools,” “frequency,” “clustering,” and “urgency.” A score of 0 to 20 indicates no LARS, 21 to 29 – minor LARS, and 30 to 42 – major LARS [11].

### Intraoperative neuromonitoring. procedure and application

**Fig. 1:**
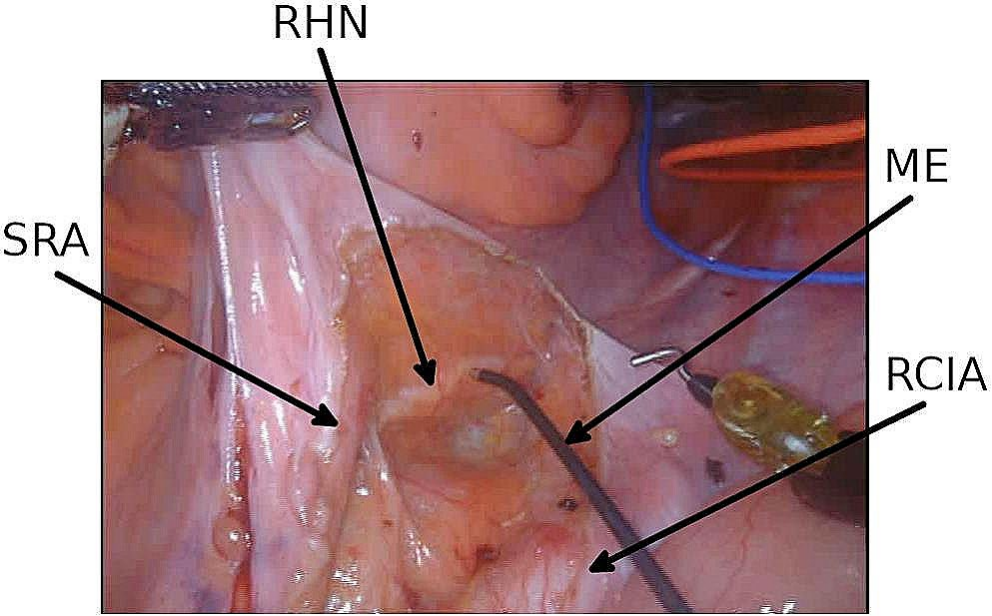
Surgical site during a low anterior rectal resection for rectal cancer with the use of neuromonitoring. Nerve stimulation using the laparoscopic hand probe (monopolar electrode) during the dissection at the pelvic inlet. SRA – superior rectal artery. RHN – right hypogastric nerve. ME – monopolar electrode. RCIA – right common iliac artery

The prototype of a new neuromonitoring system for pelvic autonomic nerve monitoring AVALANCHE^®^ NeuroNeB (Dr. Langer Medical GmbH, Waldkirch, Germany) was used. Tissue impedance was measured between two electrodes placed intraoperatively on each of the target organs, the bladder and the rectum. At the bladder, a needle electrode was inserted into the wall at the apex and a catheter electrode was inserted transurethrally (urethral sphincter). On the rectum, a needle electrode was inserted into the upper rectum and a rectal probe into the anal canal. Intraoperative neuromonitoring was then carried out by the surgeon, who stimulated the autonomic nerves with a handheld probe (Fig. 1 ). The slow contraction of the smooth muscles of the bladder and the rectum leads to a change in the muscle tissue impedance. The measured and processed voltage U(t), which was proportional to the target organs’ tissue impedance, was displayed on the AVALANCHE neuromonitor over time. This special impedance measurement was published in detail in 2022 and 2024 [5, 9, 12]. The characteristic signal shape of the impedance change was recorded and interpreted by a biomedical engineer. This method works with an inserted urinary catheter in an empty bladder, so no time-consuming bladder filling is required. The stimulation with the subsequent impedance measurement was carried out repeatedly during the surgery. As most patients underwent a low resection, the rectal probe had to be removed before the anastomosis was performed, so that the final impedance measurement was carried out only on the urinary bladder.

### Statistical analysis

The statistical analysis was performed using SPSS 21.0 (IBM Corporation, USA). The Kolmogorov-Smirnov and the Shapiro–Wilk test were used to determine whether a dataset follows a normal distribution. Mean and standard deviation describe the central tendency for normally distributed data and median and interquartile range for non-normally distributed data. Non-parametric tests were used to analyze paired samples (before and after surgery) when the differences were not normally distributed. The Wilcoxon signed-rank test was applied if the distribution of differences was symmetric and the sign test - for asymmetric distribution (each Test was two-tailed). The significance level α was set at 0.05 for all tests.

## Results

From December 2020 until November 2022, 30 patients underwent anterior rectal resection with the application of intraoperative neuromonitoring. In 28 of the 30 patients (93.3%), stimulation of the autonomic nerves exposed during dissection at the pelvic inlet induced a change in tissue impedance that could be successfully detected. Subsequently, throughout the entire procedure and finally, after extraction of the specimen, nerve stimulation could be repeatedly triggered and the corresponding response recorded, confirming the visually identified anatomical course of the nerves. Two qualified and certified colorectal surgeons performed all the surgeries, with 29 of the procedures (95%) done by one of them. A robotically assisted procedure was performed in the majority of patients (*n* = 26, 87%). One patient underwent laparoscopic resection and three patients underwent an open approach. In one patient, the robotically-assisted procedure had to be converted to an open procedure due to accidental bleeding. The patients were 66 years old on average, with a balanced gender distribution of 16 men and 14 women. The mean operating time was 393 min (SD 110 min.). None of the patients died in the first thirty days after surgery. We found a 30-day morbidity of 23% (7/30), with 3 patients suffering a severe complication ≥ 3a according to the Clavien Dindo classification (10%). One of the 24 patients with primary anastomosis developed an anastomotic leakage (4,2%). No adverse effects were observed due to the placement of the electrodes and the electric stimulation of the nerves. 18 patients received an end-to-side and 6 patients an end-to-end anastomosis. Two of the patients had a third-degree rectal prolapse, while 28 underwent resection for adenocarcinoma in the lower part of the sigmoid colon or rectum. The demographic and clinical characteristics of the patients with colorectal carcinoma are shown in Table 1. The majority of patients had rectal cancer located 12 cm or less from the anal verge (22/28; 79%). 16 patients (53%) had received neoadjuvant radiochemotherapy or radiotherapy before surgery. In almost all patients, the TME (total mesorectal excision) had excellent quality and was graded as “complete” [13]. In only one patient, the pathologist classified the TME quality as “nearly complete”.

**Table 1:** Demographic and clinical characteristics of the patients with adenocarcinoma in the lower part of the sigmoid colon or rectum (*n* = 28)

age at the time of surgery (year), median (interquartile range)	71 (15)
sex male/female	16/12
operative time (min), mean (SD)	399 (114)
ASA classification	
ASA I	0
ASA II	20
ASA III	8
ASA IV	0
Rectal resection without sphincter preservation (patients)	6
pT-category*/ ypT-category**	
pT0/ypT0	0/5
pT1/ypT1	5/2
pT2/ypT2	1/4
pT3/ypT3	6/5
pT4/ypT4	0/0
Carcinoma height from the anal verge median (interquartile range)	10 (7) cm
Available data for stapler firings (number of patients)	
- 3 magazines	5
- 2 magazines	11
- 1 magazine	11
deviating ileostomy	18
anastomotic height from the anal verge, median (interquartile range)	5 (1) cm

* pathologic T-category

** post-neoadjuvant therapy pathologic T-category

### Functional outcome

Micturition and/or defecation disorders were observed preoperatively in quite a few patients. 23% of the patients (7/30) had a significant increase in residual urine volume of more than 50 ml. Almost every second patient reported moderate (IPSS 8–19 points, 40%,) or severe symptoms (IPSS 20–35 points, 6,7%) of urinary dysfunction. Minor LARS (21–29 points) was observed preoperatively in 6 patients (20%), while 8 patients (26,7%) reported a severe defecation disorder corresponding to major LARS (30–42 points). 16 patients had 20 or fewer points (no LARS).

**Fig. 2:**
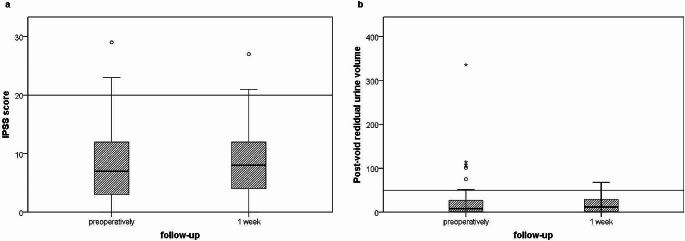
Box plots of the IPSS scores (**a**; *n* = 29) and PVR (**b**; *n* = 28) obtained before (left) and one week after surgery (right). The horizontal line, which runs parallel to the x-axis and intersects the y-axis: - in diagram **a** at 20 points, indicate the threshold for a severe IPSS. - in diagram **b** at 50 ml, indicate the threshold for a clinically significant residual urine volume

29 of the patients completed the IPSS score questionnaires in the first week after surgery, and RPV was measured in 28 of them. A summary of the two variables over time (before surgery and one week after) is shown in Fig. 2 using boxplots. The LARS score questionnaires were completed by only 4 patients one week after surgery, mainly due to the high proportion of patients who received a deviating ileostomy, so that a graphical representation does not seem appropriate.

### LARS Score

**Fig. 3:**
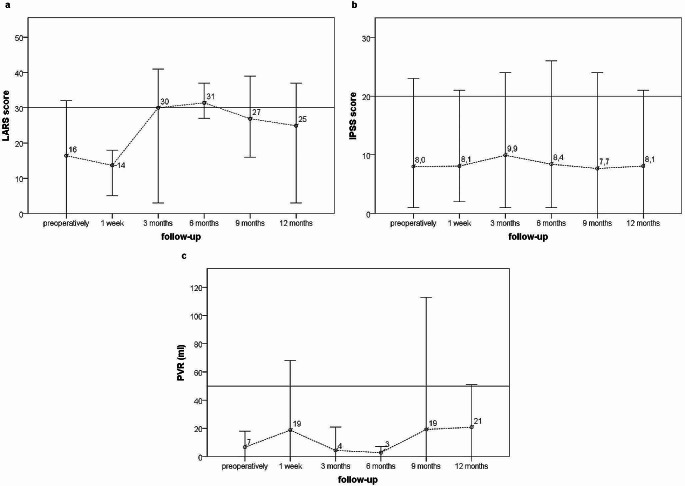
Presented is the development of the LARS score (**a**), the IPSS score (**b**) and the Post-Void Residual Urine - PVR (**c**) during the observation period in all patients who attended a final follow-up examination 12 months after surgery (*n* = 10 for LARS score and *n* = 12 for IPSS and PVR). Intraoperative findings (positive signals after stimulation) correlated with clinical results regarding urinary and fecal continence (PVR, IPSS, LARS). Each vertical line represents the observed value at the indicated follow-up examination. The upper endpoint corresponds to the highest value and the lower endpoint to the lowest. The small circle on each line represents the mean value. The horizontal line, which runs parallel to the x-axis and intersects the y-axis: - in diagram **a** at 30 points, indicate the threshold for a major LARS score. - in diagram **b** at 20 points, indicate the threshold for a severe IPSS. - in diagram **c** at 50 ml, indicate the threshold for a clinically significant residual urine volume

Two of the 12 patients with a completed follow-up still had a deviating ileostomy at the last examination and, therefore, no LARS score could be determined in these cases (*n* = 10). We observed an average increase in the LARS score of 9 points at the end of the observation period (Fig. 3 3). An exact sign test was used to compare the differences in the LARS score before and 12 months after surgery and showed statistical significance (*p =* 0.04). In 7 of these 10 patients, a deviating ileostomy had been reversed, 5 had been treated with neoadjuvant radiotherapy and the anastomosis was located on average 5.4 cm from the anal verge.

### IPSS score

The average IPSS score of the 29 patients who filled out the questionnaires in the first week after surgery was 10 points (9 points preoperatively), and severe IPSS was reported in 4 patients, two more than before the surgery (Fig. 2 ). No statistically significant deterioration was observed (Wilcoxon signed rank test, *p =* 0,7).

In the subgroup of patients with complete follow-up (*n* = 12), we compared the initial values ​​of the IPSS score with the values ​​at the final examination one year after the procedure (Fig. 3). A Wilcoxon signed rank test showed that there was no significant difference (*p =* 0,93). Two patients experienced a deterioration of more than 3 points. Only one patient had a severe IPSS with 21 points at the 12-month follow-up, after having 23 points before surgery.

### Post-void residual urine volume (PVR)

RPV was measured in 28 of the patients in the first week after surgery (Fig. 2). Before the procedure, 7 patients had an RPV of more than 50 ml (between 52 and 336 ml), whereas, in the first week after surgery, residual urine volume of more than 50 ml was measured in 4 of the patients. All other patients had no clinically relevant RPV. The highest residual urine volume measured at the last follow-up examination in the subgroup of patients with complete follow-ups (*n* = 12) was 51 ml (Fig. 3). As a PVR volume of less than 50 ml is accepted as adequate bladder emptying, no further analysis was performed.

The two patients with no response to nerve stimulation (initially and during the entire operation) had no clinically relevant increase in RPV postoperative and no LARS (LARS score up to 20 points). The IPSS score remained constant (+/- 2 Points). These patients were not present for the last follow-up examination at 12 months.

## Discussion

Up to 38% of the patients after LAR with TME are suffering from urinary incontinence, up to 46% of the patients experience bladder voiding difficulties [1, 3, 14], and 30 − 80% suffer from low anterior resection syndrome (LARS) [15]. As these disorders directly affect the patient’s quality of life, preventive strategies are becoming increasingly important. The iatrogenic injury of the pelvic autonomic nerves has an important role in the pathogenesis of functional disorders after rectal resection. During dissection at the pelvic inlet, the superior hypogastric plexus can be injured, which is located below the point of the aortic bifurcation. It divides into the left and right hypogastric nerves, which can be found, anterior and caudal to the iliac arteries. The two hypogastric nerves branch out and merge with the parasympathetic splanchnic nerves to form the inferior hypogastric plexus. An injury of the pelvic autonomic nerves is only one of the factors that can impact the complex storage and evacuation function of the bladder and rectum, but it is one of the few that can be directly influenced by the surgeon by respecting the „holy plane“ [1]. Several reports suggest better preservation of the pelvic autonomic nerves and a possible positive effect on postoperative continence and functional outcome after rectal resection when intraoperative pelvic neuromonitoring is performed based on electromyographic observation of the internal anal sphincter and manometry of the urinary bladder [7, 16]. A meta-analysis on intraoperative neuromonitoring in rectal cancer surgery from 2021 came to the similar conclusion. However, this review only included a total of 9 studies with small patient cohorts, 7 of which were conducted by the same research group and 5 included a control group [17].

The clinical investigation underlying this work was performed to investigate the feasibility of bioimpedance measurement as a new neuromonitoring method. The technical aspects of the study have already been published by our research group [5, 9, 12].

Low anterior resection syndrome (LARS) refers to a bowel dysfunction that occurs after sphincter-preserving surgery for rectal cancer and is associated with symptoms such as incontinence for flatus and/or feces, urgency, and frequent bowel movements [11]. The causes of LARS are considered to be mechanical, as the rectal reservoir is resected meaning a reduced capacity and sensation of filling, and functional, as the hypermotility of the colon and hyposensitivity to mechanical stimuli of the neorectum is caused by denervation of the colon during surgery [18–20]. Another potential cause of LARS is the dysfunction of the anal sphincter. It can be the result of a direct lesion of the anal sphincter during an ultra-low anterior rectal or an intersphincteric resection. Excision of the anal transition zone can also lead to anal sphincter dysfunction due to impaired anal sensation [18–20]. Besides intraoperative nerve damage, several other factors have proven to have a negative effect on bowel function and increase the risk of LARS after restorative rectal cancer surgery [21–23]. A short rectal remnant (< 4 cm) was found to be associated with a particularly high risk of severe bowel dysfunction [21]. The neoadjuvant radiotherapy/radiochemotherapy was identified as a strong independent risk factor for major LARS. Patients who receive combined treatment - neoadjuvant radiotherapy/radiochemotherapy and sphincter-preserving anterior rectal resection have been proven to have significantly worse bowel function than patients who have only undergone surgery [21–23]. According to a systematic review and meta-analysis with a total of 50 included studies, in addition to neoadjuvant radiotherapy, TME, anastomotic leak, and diverting stoma were associated with an increased risk of major LARS [24]. To assess the degree of bowel dysfunction we used the LARS score. The LARS score is a validated and worldwide acknowledged score that was developed to measure the severity of this condition [11, 25]. It correlates with quality of life and allows a quick clinical evaluation of the severity of LARS as well as a comparison of the symptoms caused by bowel dysfunction in different patients or the same patient at repeated assessment time points after surgery. The incidence of LARS after sphincter-preserving surgery has been reported to be up to 80% [15], whereby the above-mentioned systematic review revealed a pooled incidence of 44% for major LARS [24]. However, even patients who have not previously undergone rectal resection can exhibit symptoms typical for LARS. A Danish study investigated the prevalence of LARS in the general population and showed that 19% of women and 10% of men between the ages of 50 and 79 have symptoms consistent with major LARS. In our study population (*n* = 30), 26.7% of the patients reported a major LARS score before surgery. In the subgroup of patients who completed their 12-month follow-up, after an initial deterioration in the LARS score, a continuous improvement was observed over time. Finally, the median LARS score at the 12-month follow-up examination was 9 points higher than the median initial score before surgery (*p =* 0,04). However, the pathogenesis of LARS is multifactorial, and it must be considered that in our study population, the high-risk factors for the occurrence of LARS such as neoadjuvant radiotherapy, low anastomosis and deviated stomas were present in the majority of patients, thus deterioration of bowel function was to be expected. We performed the ileostomy reversal on average 18 weeks after the rectal resection. As known, these patients initially have a more pronounced bowel dysfunction and a poor LARS score [26–28]. It has been reported that bowel function improves significantly in the first 18 months after rectal resection [28]. The short follow-up limited to 12 months does not allow us to validate this observation in our patient population.

The rectal resection inevitably leads to morphological changes to the bowel and eventually the anal sphincter - the efferent organs responsible for fecal evacuation and continence. Due to the multifactorial etiology, a deterioration in bowel function can occur even if the pelvic autonomic nerves are preserved. This makes it difficult to assess the value of intraoperative neuromonitoring based on bowel function in a single-arm study. In contrast, urinary dysfunction is mostly caused by injury to the hypogastric nerves, as the morphological integrity of the urinary organs remains largely unaffected by rectal resection. Thus, we believe that the condition of the urinary function better reflects the integrity of the pelvic organ innervation and is considered to be the more important parameter in our study. Neoadjuvant radiotherapy has been reported to affect various aspects of urinary and sexual function [29]. Abdominoperineal resection and preoperative urinary dysfunction are considered further risk factors [14]. The risk factors mentioned were present in a considerable part of our study population. The IPSS score was obtained in 29 of the patients in the first weeks after surgery and revealed preserved urinary bladder function (*p =* 0,7). In the subgroup of patients with complete follow-ups, the IPSS score showed no substantial change at 12 months postoperatively compared with the preoperative data. The statistical test also showed no significance. Finally, a single patient had a severe IPSS with a score of 21 points at the end of their follow-up. However, this patient did not experience any deterioration as the initial IPSS score before surgery was 23 points. The residual urine volume was examined as an objective parameter for evaluating the emptying of the urinary bladder. A PVR volume of up to 50 ml indicates sufficient emptying of the bladder. For elderly individuals, even volumes between 50 and 100 ml are regarded as normal [30]. The RPV was assessed in 28 patients in the first postoperative week and, together with the analysis of the RPV in patients with complete follow-ups, revealed a preserved voiding function of the bladder, which in turn could be interpreted as a sign of preserved bladder innervation.

The available data shows no conclusive evidence for the superiority of intraoperative neuromonitoring over visual nerve identification with regard to paralysis of the inferior laryngeal nerve in adults undergoing thyroid surgery [31]. Based on this data, a substantial improvement in the functional outcome by the use of intraoperative neuromonitoring in rectal resections cannot be assumed if the procedures are performed by experienced surgeons. However, it should be considered that the functional disorders after a rectal resection are multifactorial and nerve-related complications after a thyroidectomy are probably significantly lower than after pelvic surgery, so that a direct comparison does not seem entirely appropriate. In our cohort, a signal after stimulation of the visually identified pelvic autonomic nerves could be detected in 28/30 patients (93.3%) at the beginning of the surgical dissection at the pelvic outlet. Especially the difficult preparation of the left branch could be facilitated by identification through intraoperative neuromonitoring. Repeated stimulation during the operation and especially after complete TME was able to reproduce this response, confirming the feasibility of the method. This suggest that the method may be used as a quality indicator. On the other hand, however, the postoperative functional outcome also depends on other factors. In addition, a detected signal does not necessarily have to be associated with unaltered function. This needs to be verified in larger studies.

The limitations of the study include the small number of subjects (*n* = 30) and the even smaller proportion of patients available for the evaluation of functional outcomes. Furthermore, this was a non-controlled study, which makes it difficult to assess the occurrence of multifactorial disorders such as bowel dysfunction.

## Conclusion

The feasibility of the new method for intraoperative pelvic autonomous neuromonitoring was proven in the first 30 patients.

The benefit was clear in difficult intraoperative situations, where the visualization of the pelvic nerves was challenging (obese patients, narrow male pelvis and bulky tumor). Especially the left branch of the superior plexus hypogastricus could be rapidly identified and preserved by neuromonitoring.

Intraoperative findings (positive signals after stimulation) correlated with clinical results regarding urinary and fecal continence (PVR, IPSS, LARS)

## Data Availability

No datasets were generated or analysed during the current study.
